# Editorial Announcements

**Published:** 1913-06

**Authors:** 


### Editorial Announcements

The July issue will devote considerable space to the various activities in connexion with the annual commencement and alumni meetings of the University of Buffalo, and we hope also to have similar announcements regarding Syracuse University, the only other graduate medical school in our special territory.

Owing to the unexpected demand for extra copies of the May issue, our reserve file is practically exhausted, and we shall consider it a personal favor if our readers who have no further use for this issue, will send copies to Dr. James MacLeod of Buffalo, who wishes a number on account of the obituary notice of his brother.

---

A Memorial to Field Surgeons is proposed for Gettysburg by Dr. S. Weir Mitchell of Philadelphia. Thirteen Union Surgeons were killed or wounded in this battle, and the Confederates left eighty surgeons in charge of their wounded.

